# Standard Bread

**Published:** 1923-05

**Authors:** 


### Standard Bread.

SIR,—I was glad to see the letter on this subject from "B. A. C." in your last issue, together with your editorial approval of it. Can nothing be done to induce bakers to give us really palatable and nutritious bread? No doubt they will say, as you do, that their customers love to have ill-baked, tasteless bread. But once upon a time people were content with smelly omnibuses carpeted with straw and uncomfortable "knife-boards" on the top of them. How were they weaned from these barbarities? Simply by the introduction of something better, and I have little doubt that better bread would be appreciated by large numbers of people—the lower classes would, of course, still clamour for white bread, because they think it is superior and "genteel."

Cannot the doctors, so many of whom read THE HOSPITAL AND HEALTH REVIEW, come to our help? Cannot they tell their patients who suffer from indigestion that they will never be really cured until, among other things, they eat bread which retains its goodness? Brown bread, as you say, is excellent—as a rule, but not always. What we need is a reasonable cross between white and brown, a bread which retains the best of the "offals," and is calculated, like the north-east wind (bad cess to it!) to "brace brain and sinew."

A SUFFERER FROM "LILY WHITE" BREAD.

